# Aluminium Gauze Reduces SARS-CoV-2 Viral Load in Non-Woven Masks Worn by Patients with COVID-19

**DOI:** 10.3390/idr14020030

**Published:** 2022-04-06

**Authors:** Yuto Yasuda, Satoru Mutsuo, Motoaki Hamada, Kazuo Murai, Yutaka Hirayama, Kiyoshi Uemasu, Soichi Arasawa, Daisuke Iwashima, Ken-ichi Takahashi

**Affiliations:** 1 Department of Respiratory Medicine, Kishiwada City Hospital, 1001 Gakuhara-cho, Kishiwada-shi 586-8501, Japan; kuemasu@kuhp.kyoto-u.ac.jp (K.U.); slime@mba.nifty.ne.jp (D.I.); kch-12@kishiwada-hospital.com (K.-i.T.); 2 Department of Central Clinical Laboratory, Kishiwada City Hospital, 1001 Gakuhara-cho, Kishiwada-shi 586-8501, Japan; wmutsuo@yahoo.co.jp; 3 Department of Research and Development, Dawn Inc., 1-13-5 Ayabe Bld.3F-B, Isehara-shi 259-1131, Japan; hamada@dawn-hd.co.jp; 4 Department of Engineering, Dawn Inc., 1-13-5 Ayabe Bld.3F-B, Isehara-shi 259-1131, Japan; murai-h@dawn-hd.co.jp; 5 Department of Respiratory Medicine, Kyoto University Graduate School of Medicine, 54 Kawahara-cho Shogoin Sakyoku, Kyoto 606-8507, Japan; yutabonn1016@gmail.com; 6 Department of Gastroenterology, Kishiwada City Hospital, 1001 Gakuhara-cho, Kishiwada-shi 586-8501, Japan; sarasawa@kuhp.kyoto-u.ac.jp

**Keywords:** COVID-19, aluminium, SARS-CoV-2, PCR, non-woven mask

## Abstract

Background: Aluminium reduces severe acute respiratory syndrome coronavirus 2 (SARS-CoV-2) survival in experimental settings. It is unknown whether adding an aluminium gauze to a mask reduces the SARS-CoV-2 RNA load in the mask and whether SARS-CoV-2 is detectable in the breath that permeates through such a mask in clinical settings. Methods: Patients admitted to Kishiwada City Hospital, Osaka, Japan, between July 2021 and September 2021 were enrolled in the study. Non-woven masks comprising filters with 99% viral filtration efficacy and aluminium and cotton gauzes attached to plastic collection cases were developed. All participants wore the experimental mask models for three hours. Results: Twenty-nine patients who wore the final model masks were analysed in this study. The Ct values of the nucleocapsid gene and envelope gene of SARS-CoV-2 were significantly higher in the aluminium gauze than in the cotton gauze. SARS-CoV-2 RNA was detected in the masks of 8 out of 12 vaccinated patients (66.7%). Although breath condensates were collected behind both aluminium and cotton gauzes, SARS-CoV-2 RNA was not detected in these condensates. Conclusions: Our study indicated that non-woven masks with an aluminium gauze may obstruct SARS-CoV-2 transmission in clinical settings better than non-woven masks with cotton gauzes.

## 1. Introduction

Inhalational exposure is a central route for severe acute respiratory syndrome coronavirus 2 (SARS-CoV-2) transmission [1]. Medical masks are important for preventing SARS-CoV-2 transmission [2]. However, recent evidence shows that masks cannot completely limit transmission and can only reduce exposure to viral RNA in fine aerosols by 48% [3]. The mask design should be improved to better obstruct viral transmission.

Since contaminated materials used in medical face masks can cause SARS-CoV-2 infection, it is necessary to identify new materials that prevent such contamination. Aluminium is one such promising material that decreases the survival of SARS-CoV-2. The half-life of SARS-CoV-2 survival was 2.3 h on aluminium in experimental settings [4]. However, it is unknown whether aluminium reduces SARS-CoV-2 RNA load and prevents the permeation and survival of SARS-CoV-2-infected droplets and aerosols in masks under clinical settings. Therefore, we developed an experimental mask containing an aluminium gauze and investigated SARS-CoV-2 RNA load in the mask components and in patients with coronavirus disease (COVID-19) who wore these masks.

## 2. Materials and Methods

### 2.1. Patients

Patients who were hospitalized with COVID-19 at Kishiwada City Hospital (Osaka, Japan) between 1 July 2021 and 30 September 2021 were enrolled in this study. A prototype of the experimental mask was worn by 8 patients. The final model of the mask was worn by 29 patients, who were included in the study. Relevant clinical data were collected through a retrospective review of the patients’ medical charts. Vaccination was defined as 14 days or more after the first inoculation with BNT162b2.

### 2.2. Mask Device and Analysis

**Figure 1:**
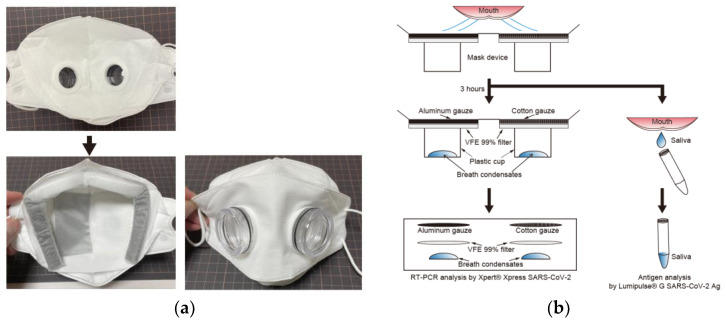
**Development of experimental mask.** The final model of the experimental mask (**a**) was composed of non-woven masks with an aluminium gauze placed in the left half, a cotton gauze placed in the right half, non-woven filters with 99% VFE, and plastic cups; (**b**) is a brief graphical summary of the experiment.

The prototype mask model was composed of a plastic sponge barricade; an outer and inner non-woven mask material with 99% viral filtration efficacy (VFE) (Tantore, Aichi; Musashino Seiki, Kanagawa, Japan), punched out for plastic case attachment; a single-layered aluminium gauze placed in the left half of the mask (DAWN, Kanagawa, Japan); a single-layered cotton gauze (Suzuran, Aichi, Japan) placed in the right half of the mask; a non-woven filter with 99% VFE (Musashino Seiki, Kanagawa, Japan) placed on both left and right halves of the mask; and plastic cases (DAWN, Kanagawa, Japan) to collect the condensation of permeated breath. The final mask model was composed of a plastic sponge barricade; an outer and inner non-woven mask material with 99% viral filtration efficacy (VFE) (Tantore, Aichi; Musashino Seiki, Kanagawa, Japan), punched out for plastic case attachment; a double-layered aluminium gauze placed in the left half of the mask (Tantore, Aichi, Japan); a double-layered cotton gauze (Suzuran, Aichi, Japan) placed in the right half of the mask; a non-woven filter with 99% VFE (Musashino Seiki, Kanagawa, Japan) placed on both left and right halves of the mask; and plastic cases (DAWN, Kanagawa, Japan) (Figure 1a). Patients wore the mask for 3 h. Immediately after 3 h, saliva was collected in a sterile tube, and each gauze and filter were cut into circles of 33 mm diameter. The SARS-CoV-2 antigen in saliva and SARS-CoV-2 RNA in gauze and filter were analysed within an hour (Figure 1b). If breath condensates were collected in the plastic cases, the entire collected fluid was measured and analysed using reverse transcription—polymerase chain reaction (RT-PCR).

RT-PCR analysis was performed using Xpert^®^ Xpress SARS-CoV-2 (Cepheid, California) on the GeneXpert^®^ instrument system (Cepheid, Sunnyvale, CA, USA) according to the manufacturer’s recommendations. The Xpert Xpress assay detected the nucleocapsid (N2) and envelope (E) genes. Briefly, the contents of the specimen collection tube and gauze or filter were mixed by rapidly inverting the tube five times. Three hundred microliters of the mixed specimen was transferred to the sample chamber of the assay cartridge. For breath condensates, all volume of breath condensate was transferred to the sample chamber of the assay cartridge. They were loaded onto the GeneXpert^®^ platform. To investigate the influence of aluminium gauze in reagents, the same size of new aluminium or cotton gauze was added to specimens in which SARS-CoV-2 RNA was detected, and RT-PCR was performed as described above. The higher limit of the Ct value was determined to be 45 cycles, according to the manufacturer’s data sheet.

Salivary SARS-CoV-2 antigen was assessed using Lumipulse^®^ G SARS-CoV-2 Ag (Fujirebio, Tokyo, Japan) on Lumipulse^®^ G 600 II as per the manufacturer’s recommendations. The higher and lower titer limits were determined to be 5000 pg/mL and 0.6 pg/mL, respectively, according to the manufacturer’s data sheet.

### 2.3. Statistical Analyses

Continuous variable data are expressed as mean ± standard deviation (SD) or median (interquartile range). The *p*-values of Ct were calculated using the Wilcoxon rank sum test. The *p*-values of patient characteristics were calculated using Student’s *t*-test, Mann–Whitney U test, or Fisher’s exact test. Correlations between salivary antigens and each Ct value were analysed using the Spearman correlation test. All statistical analyses of patient characteristics were performed using R, version 4.1.0. All statistical analyses of the experimental data were performed and visualised using GraphPad Prism, version 9.2.0 (GraphPad Software, San Diego, CA, USA). Statistical significance was set at *p* < 0.05.

## 3. Results

**Figure 2:**
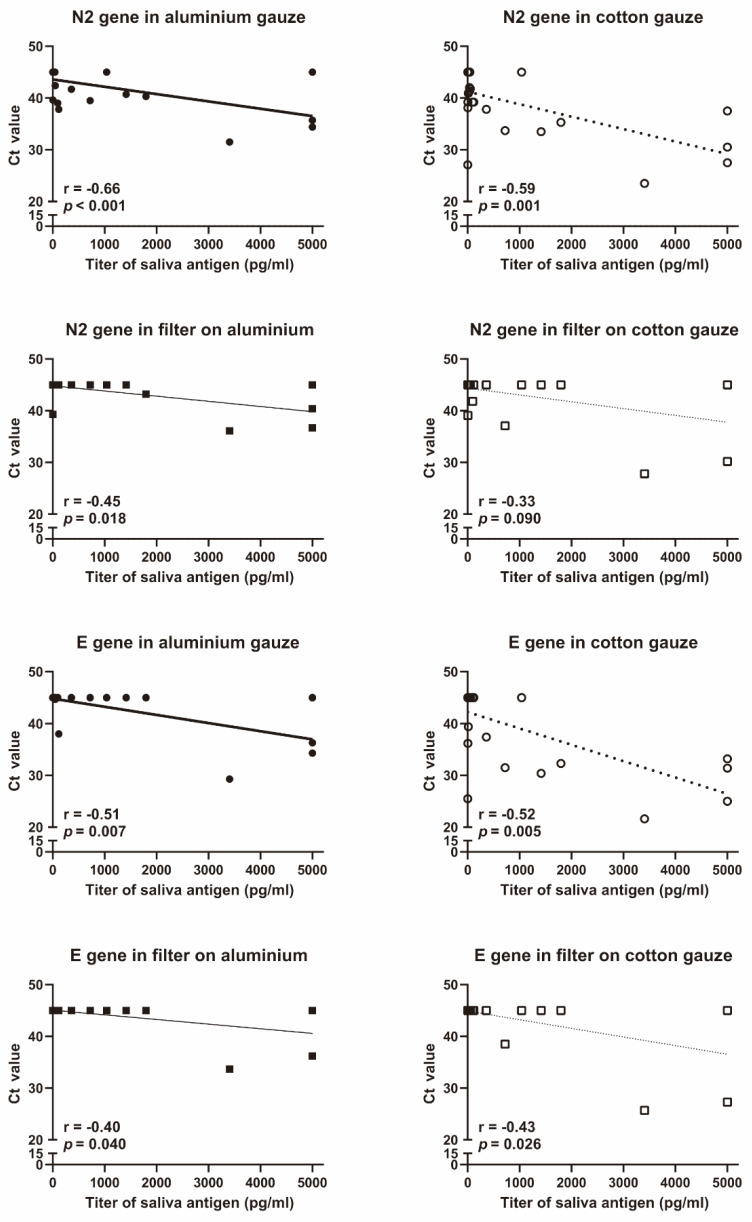
**Correlation between saliva antigen and N2 or E genes of SARS-CoV-2 in gauzes and filters.** The N2 and E genes of SARS-CoV-2 were analysed using Xpert^®^ Xpress SARS-CoV-2 (Cepheid, Sunnyvale, CA, USA). Salivary SARS-CoV-2 antigen was assessed using the Lumipulse G SARS-CoV-2 Ag (Fujirebio, Tokyo, Japan). The r and *p* values were evaluated using the Spearman rank correlation test.

**Figure 3:**
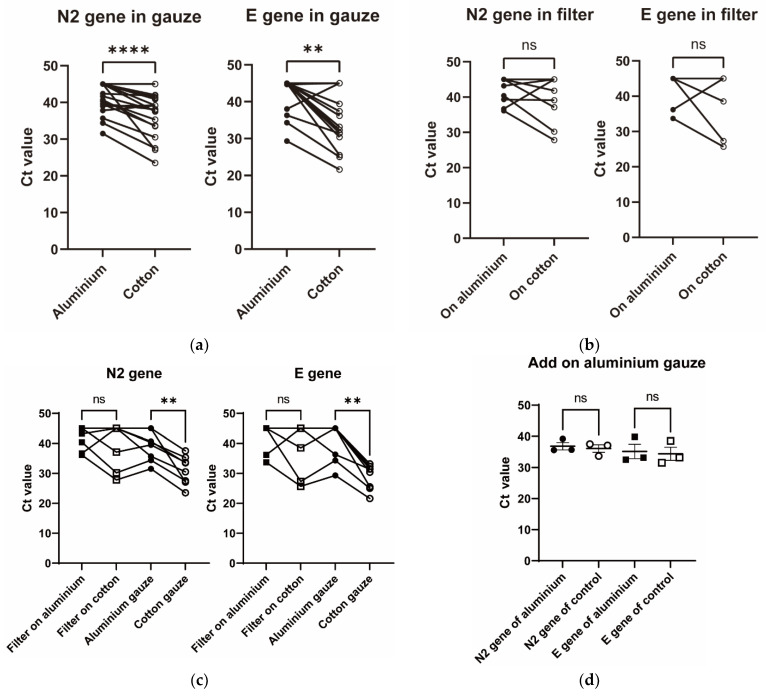
**Ct values of N2 or E genes of SARS-CoV-2 in gauzes and filters.** Ct values of N2 or E gene of SARS-CoV-2 were compared between cotton gauzes and aluminium gauzes (**a**) or between filters placed behind the cotton and aluminium gauzes (**b**). In the subgroup with a Ct value of less than 35 for either gauze or filter, Ct values of N2 or E gene of SARS-CoV-2 were compared between cotton gauzes and aluminium gauzes or between filters placed behind the cotton and aluminium gauzes (**c**). Ct values of the N2 or E gene of SARS-CoV-2 were evaluated in the filter placed behind the aluminium gauze or in the filter placed behind the cotton gauze (**d**). All *p*-values were calculated using the Wilcoxon rank sum test. **** indicates *p*-value < 0.0001. ** indicates *p*-value < 0.01. ns indicates statistically non-significant *p*-value.

Of the 29 patients who wore the final model masks, 18 had more than 10 pg/mL of SARS-CoV-2 antigen in saliva. The titer of SARS-CoV-2 antigen in saliva was negatively correlated with the Ct value of SARS-CoV-2 PCR in each gauze (Figure 2). The Ct values of the N2 and E genes of SARS-CoV-2 were significantly higher in the aluminium gauze than in the cotton gauze (Figure 3a). The Ct values of the N2 and E genes of SARS-CoV-2 tended to increase, but not significantly, in the filter placed behind the aluminium gauze than in the filter placed behind the cotton gauze (Figure 3b). Since many of the Ct values were greater than 35, we analysed the Ct values in the data where the Ct value of either gauze or filter was less than 35. All eight patients analysed had Ct values less than 35 of E gene in the cotton gauze. In this subgroup analysis, the Ct values of the N2 and E genes of SARS-CoV-2 were significantly higher in the aluminium gauze than in the cotton gauze (Figure 3c). The influence of aluminium gauze in PCR reagents was not observed when new aluminium gauze or cotton gauze was added to the specimens in which SARS-CoV-2 RNA was detected (Figure 3d).

The median time from onset to experiment was 6 days. Thirty-eight percent of patients had cough that required antitussives. The patients’ characteristics according to whether SARS-CoV-2 RNA was detected in the gauzes or filters are summarized in Table 1. Regardless of the detection of SARS-CoV-2 RNA, all patients were cured with or without treatment. The shorter the period from onset to experiment, the more SARS-CoV-2 RNA was detected. High inflammation during hospitalization and hypoxia was not related to the detection of SARS-CoV-2 RNA. A total of 8 of the 12 vaccinated patients (66.7%) had SARS-CoV-2 RNA in the masks. Two weeks or more had passed since the second vaccination in 10 of the 12 vaccinated patients. No adverse events, including contact allergies, were observed.

**Figure 4:**
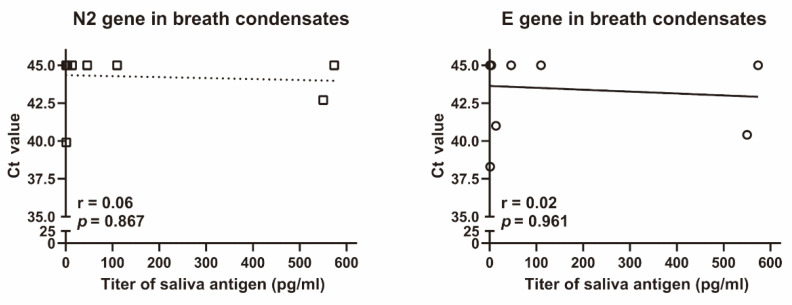
**Correlation between saliva antigen and N2 or E genes of SARS-CoV-2 in breath condensates.** Ct values of N2 and E genes of SARS-CoV-2 in breath condensates were analysed using Xpert^®^ Xpress SARS-CoV-2. Salivary SARS-CoV-2 antigen was assessed using the Lumipulse G SARS-CoV-2 Ag. The r and *p* values were evaluated using the Spearman rank correlation test.

Finally, we investigated whether breath condensates collected through a gauze and filter with 99% VFE contained SARS-CoV-2 RNA. Breath condensates were collected in the plastic cases of all eight prototype non-woven masks. The amount of breath condensate was 178 ± 95 µL (mean ± SD). Two of the eight cases contained the N2 gene of SARS-CoV-2, whereas three of the eight cases contained the E gene of SARS-CoV-2 in the breath condensates (Figure 4). Based on these results, we collected breath condensate in the final mask models. In eight masks, breath condensates were found in the plastic cases placed behind the aluminium gauze, whereas in three masks, the condensates were found in the plastic cases placed behind the cotton gauze; SARS-CoV-2 RNA was not detected in these breath condensates.

## 4. Discussion

To the best of our knowledge, this is the first report investigating the efficacy of aluminium gauze in reducing SARS-CoV-2 RNA load in masks worn by patients with COVID-19 in clinical settings. Aluminium gauze is readily available and cost-effective. Contact allergies to aluminium are rare (0.9%), and the safety of aluminium gauze is guaranteed [5]. Non-woven masks with aluminium gauze may be better for controlling the spread of SARS-CoV-2.

Numerous researchers have studied several materials to inactivate SARS-CoV-2 [6]. Copper iodide nanoparticles, copper-silver nanohybrids, silver nanocluster/silica composites, titanium dioxide, and aluminium reduce SARS-CoV-2 survival in experimental settings [4,7,8,9]. It is not known which materials are better at decreasing SARS-CoV-2 survival in clinical settings. In addition, the mechanism by which aluminium causes antiviral activity is unknown. Further research is needed to determine the optimal material for antiviral activity and to elucidate the underlying mechanisms.

Our study indicated that the shorter the period from disease onset to the experiment, the more SARS-CoV-2 RNA was detected. However, a high inflammatory reaction and hypoxia did not increase the detection of SARS-CoV-2 RNA. This suggests that the number of days since disease onset is important for transmission.

Vaccination may reduce the transmission of SARS-CoV-2 [10]. It is unclear whether vaccinated patients infected with SARS-CoV-2 will transmit the virus like non-vaccinated patients with COVID-19. Our data suggest possible transmission of SARS-CoV-2 because of the high detection of SARS-CoV-2 RNA in the masks of vaccinated patients. Therefore, vaccinated persons should also wear masks to prevent SARS-CoV-2 transmission.

It is not known whether aerosols that permeate through non-woven masks with 99% VFE can contain SARS-CoV-2 RNA. Several studies have demonstrated that expired breath contains SARS-CoV-2 RNA or antigen [11,12,13,14,15,16]. Ours is the first study to detect SARS-CoV-2 RNA in expired breath filtered with a non-woven mask with 99% VFE. This raises the possibility of SARS-CoV-2 transmission, regardless of wearing a non-woven mask. Although in our study, non-woven masks with two gauze layers accumulated a few expired breath condensates, SARS-CoV-2 RNA was not detected in these breath condensates. Therefore, a non-woven mask with a double layer of gauze might be better for controlling SARS-CoV-2 transmission.

This study had several limitations which need to be considered. First, it was a small single-center study, and the in-patient setting reduced conversation and differed from everyday life. A higher viral load of SARS-CoV-2 caused by conversation may influence the reduction in efficacy of aluminium gauze. A larger study in the community is required. Second, the Ct values were above 40 for most patients. Although the Xpert Xpress assay detects Ct values less than 45, high Ct values may result in false positives. Therefore, the present data should be confirmed in COVID-19 patients with Ct values less than 40. Third, although the use of an aluminium gauze reduced SARS-CoV-2 viral load, it is unclear whether aluminium gauze reduces SARS-CoV-2 transmission. A basic experiment to replicate SARS-CoV-2 isolated from aluminium gauze may be helpful. Fourth, the filter on the side of the aluminium gauze sometimes contained SARS-CoV-2 RNA. It is unclear how and to what extent aluminium gauze reduces the SARS-CoV-2 viral load in exposed patients. Finally, it is not clear whether mutation of SARS-CoV-2 influences the efficacy of aluminium gauze. Therefore, it is necessary to conduct further research and to evaluate the effect of the mutational state of SARS-CoV-2.

## 5. Conclusions

Our study indicates that aluminium gauze reduces SARS-CoV-2 RNA load in non-woven masks in patients with COVID-19. Non-woven masks with aluminium gauze may reduce SARS-CoV-2 transmission and may help suppress outbreaks of COVID-19.

## Figures and Tables

**Table 1:** Patient characteristics.

	SARS-CoV-2 Positive PCR *n* = 17	SARS-CoV-2 Negative PCR *n* = 12	*p* Value
Age, year-old, (mean ± SD)	54.3 ± 20.1	53.6 ± 10.6	0.912
Sex (F/M)	5/12	5/7	0.774
BMI, mg/m^2^, (median [IQR])	26.10 [22.60, 29.90]	24.90 [22.27, 29.97]	0.825
WBC, /µL, (median [IQR])	4490.00 [3940.00, 5970.00]	5100.00 [3505.00, 6195.00]	0.757
CRP, mg/dL, (median [IQR])	1.51 [0.85, 5.72]	4.94 [2.24, 8.92]	0.170
IL-6, pg/mL, (median [IQR])	10.70 [9.20, 22.60]	36.05 [11.62, 72.17]	0.223
Fever at hospitalization (Yes/No)	11/6	8/4	1.000
WHO progression scale at hospitalization (4/5)	12/5	4/8	0.108
Remdesivir treatment	4/13	7/5	0.130
REGN-COV2 treatment	14/3	4/8	0.022
Infiltrates (>50%) in chest X-ray (Yes/No)	0/17	2/10	0.163
Cough that requires antitussives (Yes/No)	6/11	5/7	1.000
Vaccination (Yes/No)	8/9	4/8	0.703
Time from onset to experiment (median [IQR])	5.00 [3.00, 6.00]	8.50 [6.50, 10.25]	0.010

SD, standard deviation; IQR, interquartile range; BMI, body mass index; WBC, white blood cell; CRP, c-reacting protein; IL-6, interleukin-6; WHO, World Health Organization.

## Data Availability

The datasets generated and/or analysed during the current study are available from the corresponding author on reasonable request.
